# Double burden of malnutrition and associated factors among mother–child pairs at household level in Bahir Dar City, Northwest Ethiopia: community based cross-sectional study design

**DOI:** 10.3389/fnut.2024.1340382

**Published:** 2024-02-20

**Authors:** Solomon Mekonnen, Dereje Birhanu, Yonatan Menber, Zenebe Abebe Gebreegziabher, Mahider Awoke Belay

**Affiliations:** ^1^North Gondar Zone Health Office, Debark, Ethiopia; ^2^Department of Nutrition and Dietetics, School of Public Health, College of Medicine and Health Sciences, Bahir Dar University, Bahir Dar, Ethiopia; ^3^Department of Epidemiology and Biostatistics, School of Public Health, Asrat Woldeyes Health Science Campus, Debre Berhan University, Debre Berhan, Ethiopia; ^4^Department of Public Health, College of Medicine and Health Science, Injibara University, Injibara, Ethiopia

**Keywords:** double burden, malnutrition, mother–child pairs, household, Bahir Dar, Ethiopia

## Abstract

**Introduction:**

The double burden of malnutrition refers to the simultaneous presence of under nutrition and overweight, obesity, or diet-related non-communicable diseases which might occur at the population, household, and individual level. The simultaneous presence of overweight/obese mothers with undernourished children in the same household, as well as overweight children with underweight mothers, holds particular significance. This phenomenon primarily impacts low-income and middle-income countries. The prevalence of double-burden malnutrition at the household level has increased significantly in sub-Saharan African countries. However, there is limited knowledge regarding the extent and factors associated with the double burden of malnutrition among mother–child pairs in Ethiopia. Consequently, the objective of this study was to assess the prevalence and determinants of the double burden of malnutrition among mother–child pairs at the household level in Bahir Dar City, Ethiopia.

**Method:**

In the year 2021, a community-based cross-sectional study design was employed among 702 mother–child pairs in Bahir Dar City from February 28 to March 23. A multistage sampling technique was used to identify study participants who were interviewed using an interviewer-administered questionnaire. The nutritional status of children was evaluated using WHO Anthro Software. To determine the strength of the association, multivariable logistic regression analysis was performed, and adjusted odds ratios with their respective 95% confidence intervals were computed.

**Results:**

The prevalence of the double burden of malnutrition among mother–child pairs was 14.5% (95% CI: 12.8, 15.7%}. Participants who were in the richest wealth index were 2.72 {AOR = 2.72, 95% CI 2.01, 5.63} times more odds of double burden of malnutrition than the poorest. The odds of the double burden of malnutrition among children who had high dietary diversity decreased by 63% {AOR = 0.37, 95% CI 0.22, 0.61} than low dietary diversity. Food secure households were 1.96 {AOR = 1.96, 95% CI 1.13, 3.39} times more likely to have the double burden of malnutrition than food insecure households. The odds of the double burden of malnutrition among mothers who completed college and above decreased by 74% {AOR = 0.26 95% CI 0.121, 0.549} than those unable to read and write.

**Conclusions and recommendation:**

The magnitude of the double burden of malnutrition was lower than the Ethiopian Demographic and Health Survey. Wealth index, dietary diversity, food security, and educational status were significantly associated with the double burden of malnutrition. Therefore, it is recommended to implement public health interventions that target the identified associated factors in order to reduce the burden of double malnutrition.

## Introduction

The term malnutrition encompasses two primary categories of conditions. The first category is “under nutrition,” which refers to deficiencies in energy and essential micronutrients, leading to conditions such as stunting, wasting, and underweight. The second category is “over nutrition,” which includes conditions such as overweight, obesity, and diet-related non-communicable diseases (NCDs) (1). Nowadays many countries are facing a double burden of malnutrition (DBM), a coexistence of under nutrition and overweight, obesity, or diet-related non-communicable diseases, affecting most low-income and middle-income countries (LMICs) (2, 3). The simultaneous presence of overweight/obese mothers with undernourished children, as well as underweight mothers with overweight children, within the same household, is a significant and noteworthy phenomenon. This phenomenon highlights the complex and multifaceted nature of malnutrition, where different forms of malnutrition can coexist within a single-family unit. Understanding and addressing this double burden of malnutrition is crucial for developing effective interventions and policies to improve the health and well-being of both mothers and children (4).

The global burden of the double burden of malnutrition (DBM) was 16% which is highly prevalent in Sub-Saharan countries (5). The DBM in households ranged from 1.71 to 17.12% in African countries (6), and 23% from further analysis of 2016 Ethiopian Demographic and Health Survey data (EDHS) (7). The double burden of malnutrition at the household level among mother–child pairs was revealed high in different studies such as 30.6% in Indonesia (8), 15.7% in Palestine (9), 6% in India (10), 24.4% in North Africa (11), and 9% in Addis Ababa and the rural district of Kersa, Ethiopia (12).

Maternal and child under nutrition is responsible for over 10% of the global disease burden, while being overweight/obese leads to at least 2.6 million deaths annually (13). The estimated effects of stunting, severe wasting, and intrauterine growth restriction together were responsible for 2.2 million deaths and 21% of disability-adjusted life-years (DALYs) for children younger than 5 years (14). Nutrition-related factors contribute to approximately 45% of deaths in children aged less than 5 years mainly due to under nutrition (15). Maternal obesity leads to gestational diabetes, pre-eclampsia, hemorrhage, and a higher risk of neonatal and infant death (16–19). In addition, 44% of the diabetes burden, 23% of the ischemic heart disease burden, and between 7 and 41% of certain cancer burdens are attributable to overweight and obesity (20).

The emergence of the double burden of malnutrition can be attributed to a series of global changes known as nutritional transition, demographic transition, and epidemiological transition. These transitions encompass shifts in dietary patterns, population dynamics, and disease profiles that have occurred worldwide. These changes have contributed to the coexistence of under nutrition and over nutrition within populations, leading to the complex issue of the double burden of malnutrition (21–24). These life-course trajectories such as rapidly changing diets, norms of eating, and physical activity patterns, and ecological factors such as pathogen burden and extrinsic mortality risk lead to DBM (25).

The World Health Organization (WHO) proposed a roadmap to tackle the DBM through so-called “double-duty actions” (26). The Global Nutrition Report has committed to eradicating all forms of malnutrition by 2030. Maternal–infant and young child nutrition (MIYCN) initiatives align with the Global Action Plan on Non-Communicable Diseases (NCDs), which includes targets to reduce obesity and other risk factors associated with non-communicable diseases (NCDs). These efforts work in tandem to address the multifaceted challenges of malnutrition and promote the health and well-being of mothers, infants, and young children worldwide (13, 27). The Ethiopian government’s obligation to end child under nutrition by 2030 has stepped forward with the recently developed National Food and Nutrition Policy (28). However, malnutrition is one of the major public health concerns worldwide with a significant increase in Sub-Saharan countries including Ethiopia (29–38). The current study was conducted in the year 2021, and holds significance as it aims to assess the double burden of malnutrition and its associated factors among mother–child pairs in Bahir Dar City, located in Northwest Ethiopia. The study provides valuable evidence and insights into new findings, which can be utilized by relevant stakeholders and organizations to develop targeted prevention strategies for addressing the double burden of malnutrition at the household level.

## Methods and materials

### Study setting

The study was conducted in Bahir Dar City. It is the capital city of the Amhara region and 565 KM away from Addis Ababa, the capital city of Ethiopia. Based on the Bahir Dar City health administration report the total population of Bahir Dar City was estimated to be 389,137, from this 198,459.87 are females and 91,758.5 are women under the reproductive age group. The number of under-five children was estimated to be 52,689.14 and of these 50,120.8 were 6–59 months old (39).

### Study design, study period, population, and eligibility criteria

From February 28 to March 23, 2021, a community-based cross-sectional study was conducted to assess both the outcome status and exposure status simultaneously. The source population consisted of all mothers with children aged between 6 and 59 months. The study population was defined as mothers with children aged between 6 and 59 months residing in randomly selected sub-cities and Kebeles of Bahir Dar City. The inclusion criteria for the study were mothers with children aged between 6 and 59 months who had been living in Bahir Dar City for more than 6 months. However, pregnant mothers were excluded from the study.

### Sample size determination and sampling techniques

The sample size was determined using the following single population proportion formula, n=Zα22P1−Pd2 , where margin error = 4%, The proportion (P) of the coexistence of overweight/obese mothers with stunted children = 23% (7), 1−*p* = 1–0.23 = 0.77, Za2=1.96at95%confidenceinterval .


$$n=\frac{{\left(1.96\right)}^{2}\left(0.23\left(0.77\right)\right)}{0.04^{2}}=425$$

Since our sampling technique is multistage sampling, a design effect of 1.5 was considered: The sample size becomes: 425*1.5 = 638.

Finally, after we adjust for a non-response rate of 10% the final sample size becomes 637 + 637(0.1) =638 + 63.8 = 701.8 ~ 702.

This study employed a two-stage random sampling technique. Initially, three sub-cities were randomly selected from the six sub-cities in Bahir Dar City. Subsequently, six Kebeles (two from each selected sub-city) were chosen at random using the lottery method. The households within each selected Kebele were sampled using a systematic random sampling approach. After randomly selecting the first household from two households, a sampling interval of two was applied to select subsequent households. If mothers had multiple children, one child was randomly chosen for the assessment of the DBM.

### Data collection tools and procedures

Prior to the actual data collection, a pre-test was conducted among 5% (*n* = 35) of the sample size from Kebeles that were not selected. The data were gathered using interviewer-administered questionnaires, which were adapted from various literature sources. These questionnaires were derived from reports such as EDHS (40), FANTA, and FAO (41), and previous research studies that had conducted similar investigations (15, 29, 42–47), with slight modifications made as necessary. The data collection process was carried out by eight experienced BSc nurses, who were supervised by two Public Health professionals.

Anthropometric measurements were employed to collect data from both mothers and children. For children under 2 years of age, their length was measured while in a recumbent position. The mothers’ body mass index (BMI) was calculated based on their height and weight. Sliding boards were used for length measurement, while digital weight scales were utilized for weight measurement. For children aged 2–5 years and mothers, their height was measured in a standing position with the Frankfurt plane, ensuring that the knees, heels, buttocks, and shoulder blades touched the vertical surface of the stadiometer. The weight of children under 2 years old was measured using a weighing sling (spring balance) after removing their shoes and heavy clothing. A digital weight scale was employed for children aged 2–5 years and mothers to measure their weight.

Asking the mother if the child had eaten anything from any of the normal seven food groups the day before, without imposing any minimum consumption requirements, was how dietary variety was determined. The seven food classes were cereals, roots, and tubers; legumes and nuts; dairy products except breast milk; flesh foods (meat, fish, fowl, or organ meats); eggs; vitamin A-rich fruits and vegetables; and other fruits and vegetables. A scale of 0–7 was used to calculate the dietary diversity score (DDS). Children were deemed to be adequate if they were fed at least four of the seven food groups throughout the reference period (48).

Minimum Dietary Diversity for a Woman (MDD-W), a dichotomous indicator/tool created by the FAO, was used in this study to quantify the dietary diversity of mothers. Ten food groups in total were taken into consideration: all starchy staples; pulses (beans, peas, and lentils); nuts and seeds; dairy products; flesh foods (including organ meat); eggs; vitamin A-rich dark green and leafy vegetables; other vitamin A-rich vegetables and fruits; and other vegetables and fruits. Every woman was classified as having adequate (>5 food groups) or not adequate (≤5 food groups) based on the Minimum Dietary Diversity Score (MDDS) that was computed for her during the previous 24 h (41).

### Data quality control

The data collection instrument was initially developed in English and subsequently translated into Amharic. To ensure accuracy, the translated version was then translated back into English. The data collectors and supervisors underwent a two-day training program prior to the commencement of data collection. Any necessary adjustments were made based on the training feedback. The face validity of the tool was assessed by five nutrition experts to ensure its appropriateness and relevance. To minimize missing data, supervisors reviewed each questionnaire on a daily basis, verifying and cross-checking all recorded information against the data collectors’ entries.

### Data processing and analysis

The collected data underwent a series of steps to ensure quality and prepare it for analysis. These steps included checking for completeness, compiling the data, coding it, and subsequently entering it into EpiData 3.1. The data was then exported to SPSS version 25.0 software for further analysis. For assessing the nutritional status of children, height-for-age, weight-for-age, and weight-for-height Z-scores were calculated using WHO Anthro version 3.2.2 software.

On the other hand, the mother’s nutritional status was evaluated by calculating the body mass index (BMI) (49). In the logistic regression model, the outcome variable was the double burden of malnutrition (DBM), a dichotomous variable labeled “1” for having DBM and “0” for not having DBM or normal status. Thus, those who had a DBM either due to the mother being overweight/obese (>BMI 25–29.9 kg/m2 overweight and ≥ 30 BMI obese) and the child being undernourished with stunting/wasting/underweight (<-2SD Z-scores) or due to the child being obese/overweight (> + 2SD Z-scores) in comparison with the underweight mothers (<BMI 18.5 kg/m2).

In the analysis, variables that demonstrated a value of *p* of ≤0.25 in the bivariable logistic regression were selected for inclusion in a multivariable binary logistic regression model. This step was undertaken to account for the potential impact of confounding variables. These variables were included in the multivariable model, enabling us to assess the individual effects of each variable while controlling for the effects of other variables. Variables with a value of *p* of <0.05 after the multivariable analysis were considered to have a significant association with the outcome variable. The degree of association between variables was measured using adjusted odds ratios, accompanied by their corresponding 95% confidence intervals. The fitness of the model was assessed using the Hosmer-Lemeshow goodness-of-fit test, which yielded a value of *p* of 0.59, indicating that the model fits the data well as the value of p is greater than 0.05. Additionally, a likelihood ratio test was conducted to compare the null model with the full model. The full model was selected due to its higher likelihood ratio.

To ensure the independence of observations, the intraclass correlation coefficient was calculated. The coefficient was found to be below 10%, indicating that the observations were independent. To evaluate the assumption of multicollinearity, the standard errors of the variables were examined. No issues of multicollinearity were detected as the standard errors were below two for all variables.

### Operational definitions

#### DBM

In this study, participants were categorized as experiencing a double burden of malnutrition if either the mother was overweight/obese and the child was undernourished (with stunting, wasting, or underweight), or if the mother was underweight and the child was overweight (1).

#### Under nutrition

Mothers body mass index (BMI) ≤ 18.5 kg/m2 (49).

#### Overweight

Mothers BMI from 25–29.9 kg/m2 (49).

#### Obesity

Mothers BMI ≥30 kg/m2 (49).

#### Stunting

Height for age value less than minus two (−2 SD) standard deviations of WHO child growth standard (50).

#### Wasting

Weight-for-height value less than minus two (−2 SD) standard deviations of WHO child growth standard (50).

#### Underweight

Weight-for-age value less than minus two (−2 SD) standard deviations of WHO child growth standard (50).

#### Overweight

Weight-for-height more than plus two (+2 SD) standard deviation of the WHO child growth standards (50).

#### Food insecurity

Food secure score (0–1), mildly food insecure (2–8), moderately food insecure (9–15), and severely food insecure (16–27) (51).

#### Maternal dietary diversity

Mothers who consumed ≤5 out of 10 food groups were considered to have low/inadequate dietary diversity, while the mothers who consumed ≥6 out of 10 food groups were considered to have a high dietary diversity score (52).

#### Child dietary diversity

A child who consumes less than four out of seven food groups was categorized as having low (inadequate) dietary diversity, while those who consume four or more were considered to have high (adequate) dietary diversity (53).

#### Sedentary behavior

Mother site more than >9 h/day (54).

## Results

### Socio-demographic and socioeconomic characteristics of the respondents

From a total of 702 mother–child pairs, 661 have participated in the study with a 94.2% response rate. The number of female children was 386 (58.4%). The number of children whose age was >2 years was 385(58.2%). About 373 (56.4%) of the mothers were in the age group of 25–34 years while 196 (29.7%) were age ≥ 35 years. Around 254 (38.4%) of the mothers were housewives, 146 (22.1%) were private employees, and 60 (9.1%) were daily laborers. More than half of the households had ≤5 family members. Concerning the wealth index 264(39.9%) of participants were the poorest, while 101(15.3%) were the richest (Table 1).

**Table 1:** Socio-demographic and socio-economic characteristics of mother–child pairs in Bahir Dar City, Northwest Ethiopia (*N* = 661).

Variables		Frequency	Percent (%)
Sex of the child	Male	275	41.6
	Female	386	58.4
Age of the child	≤2 years	276	41.8
	>2 years	385	58.2
Age of the mother	15–24	92	13.9
	25–34	373	56.4
	≥35	196	29.7
Religion	Orthodox	397	60.1
	Muslim	238	36.0
	Protestant	26	3.9
Marital status	Married	585	88.5
	Single	25	3.8
	Divorce	31	4.7
	Widowed	20	3.0
Educational status of the mother	Unable to read and write	84	12.7
	Able to read and write	38	5.8
	Primary	111	16.8
	Secondary	207	31.3
	College and above	221	33.4
Occupation status of the mother	Housewife	254	38.4
	Private employee	146	22.1
	Government employee	156	23.6
	Merchant	45	6.8
	Daily laborer	60	9.1
Occupation status of the husband	Government employee	210	35.9
	Merchant	259	44.3
	Daily laborer	59	10.1
	Private employee	57	9.7
Family size	≤5	397	60.1
	>5	264	39.9
Wealth index	Poorest	132	20.0
	Poor	132	20.0
	Middle	128	19.3
	Rich	168	25.4
	Richest	101	15.3

### Maternal and child characteristics

Nearly 205 (31.0%) of the children had low dietary diversity. Dietary diversity of the mothers was inadequate for 292 (44.2%). Most of the participants 578 (87.4%) sit for ≤540 min per day (Table 2).

**Table 2:** Maternal and child characteristics of the double burden of malnutrition among mother–child pairs in Bahir Dar City, Northwest Ethiopia (*N* = 661).

Variables		Frequency	Percent (%)
Sedentary behavior (sitting time)	≤540 min/day	578	87.4
	>540 min/day	83	12.6
DDS of mother	Low/inadequate(≤5)	292	44.2
	High/adequate(≥6)	369	55.8
Birth weight (Kg)	Low(<2.5)	148	22.4
	Normal (2.5–3.99)	505	76.4
	High(≥4.0)	8	1.2
DDS of child	Low/inadequate(<4)	456	69.0
	High/adequate(≥4)	205	31.0

### Food security status

**Figure 1:**
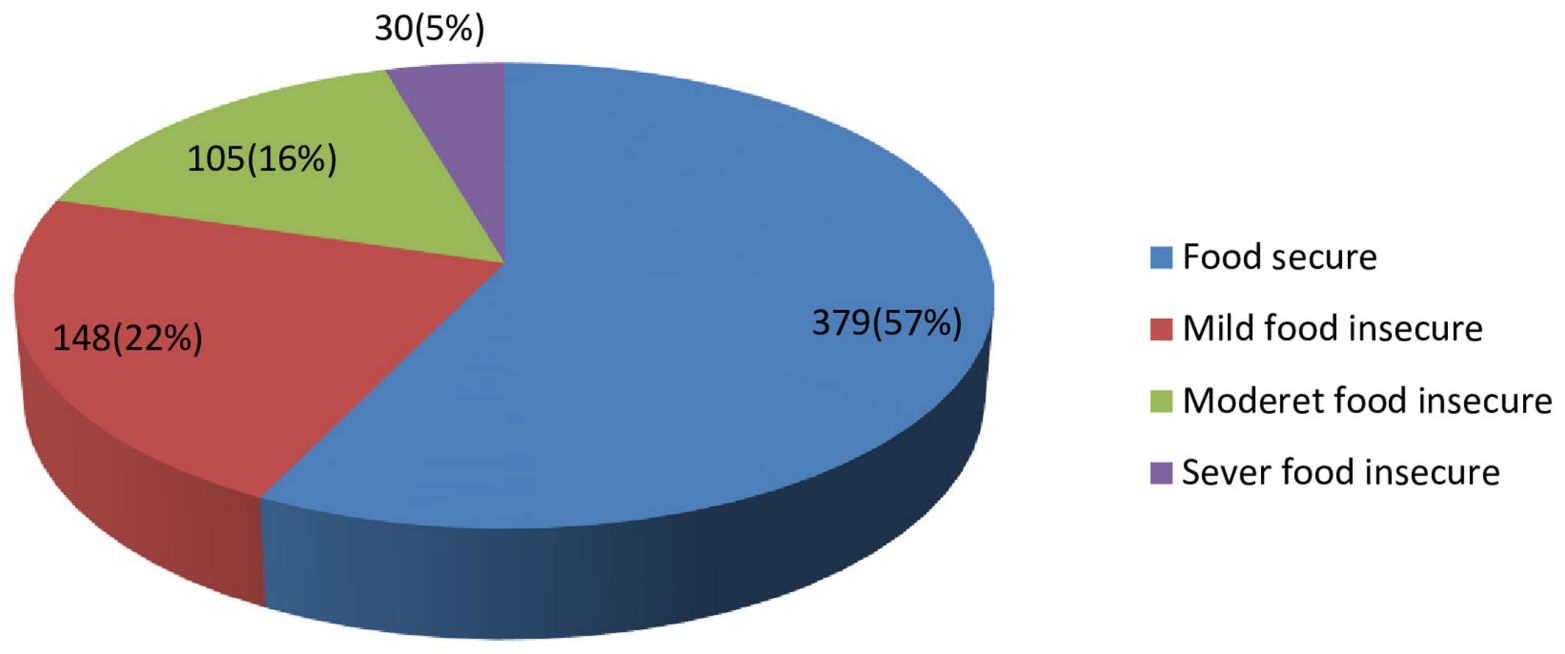
Household food security characteristics of the DBM among mother-child (instead mother to child) pairs in Bahir Dar City, Northwest Ethiopi (*N* = 661).

A total of 379 (57.25%) participants of the households had food security, while, 30(4.5%) were sever food insecure (Figure 1).

### Prevalence of double burden of malnutrition

The overall double burden of malnutrition was 14.5 95% CI (12.8 15.7%). Among this, 11.3% (95% CI: 10.51, 12.09%) of mothers were overweight/obese and the child was undernourished (with stunting, wasting, or underweight), and 3.2% were underweight mothers and obese/overweight child (Table 3).

**Table 3:** Nutritional statuses of mothers and children and the prevalence of the double burden of malnutrition among mother–child pair in Bahir Dar City, Northwest Ethiopia (*N* = 661).

Variables	Categories	Frequency	Percent (%)
Stunting	Yes	210	31.8
	No	451	68.2
Wasting	Yes	57	8.6
	No	604	91.4
Underweight	Yes	85	12.9
	No	576	87.1
underweight mothers	Yes	97	14.7
	No	564	85.3
Normal	Yes	415	62.8
	No	246	37.2
Overweight/obese mothers	Yes	149	22.5
	No	512	77.5
Overweight/obese mothers with stunting child	Yes	31	4.7
	No	630	95.3
Overweight/obese mothers with wasting child	Yes	19	2.9
	No	642	97.1
Overweight/obese mothers with underweight child	Yes	28	4.2
	No	633	95.8
Overweight/obese children with underweight mothers	Yes	21	3.2
	No	640	96.8
Overall double burden of malnutrition	Yes	96	14.5
	No	565	85.5

### Factors associated with the double burden of malnutrition among mother–child pair

In the bivariable binary logistic regression analysis, several variables showed a significant association (with a value of *p* ≤0.25) with the double burden of malnutrition. These variables included household food security, wealth index, child’s dietary diversity score (DDS), mother’s age, and mother’s educational status. However, In the multivariable binary logistic regression analysis, subsequent to adjusting for confounding factors, notable associations were observed between the double burden of malnutrition and household food security, wealth index, children’s dietary diversity status, and mother’s educational status. These variables retained their significance even after controlling for potential confounders. The confounding factors taken into account for the multivariable analysis included variables such as household food security, wealth index, mother’s dietary diversity score (DDS), child’s dietary diversity score (DDS), mother’s age, and mother’s educational status. The analysis involved controlling for the effects of these variables on one another to accurately interpret their individual effects. Participants from food-secured households had 1.96 times higher odds of experiencing the double burden of malnutrition compared to households that were food insecure (AOR = 1.96, 95% CI 1.13, 3.39). Similarly, participants from the richest households had 2.72 times higher odds of experiencing the double burden of malnutrition compared to those from the poorest wealth index (AOR = 2.72, 95% CI 2.01, 5.63). The odds of experiencing the double burden of malnutrition among children with high dietary diversity decreased by 63% compared to those with low dietary diversity (AOR = 0.37, 95% CI 0.22, 0.61). Furthermore, the odds of the double burden of malnutrition among mothers who completed college and above decreased by 74% compared to those who were unable to read and write (AOR = 0.26, 95% CI 0.121, 0.549) (Table 4).

**Table 4:** Factors associated with the DBM (overweight/obese mothers with underweight/stunted/wasted children) or (underweight mothers with overweight children) in Bahir Dar City, Northwest Ethiopia (*N* = 661).

Double burden of malnutrition					
Variables	Categories	Yes	No	COR (95%CI)	AOR (95%CI)
Mother’s age	15–24	6	86	1	1
	25–34	45	328	1.97 (0.81, 4.76)	1.82 (0.78, 5.22)
	> = 35	45	151	4.27(1.75, 10.42)	2.53 (0.93, 6.86)
Child’s DDS	Low(<4)	45	160	1	1
	High (≥4)	51	405	0.45 (0.29, 0.70)	0.37 (0.22, 0.61)*
Mother’s education status	Unable to read and write	20	64	1	1
	Able to read and write	11	27	1.30 (0.55, 3.10)	1.06 (0.40, 2.81)
	Primary education	19	92	0.66 (0.33, 1.34)	0.59 (0.41, 1.95)
	Secondary education	27	180	0.48 (0.25, 0.92)	0.46 (0.27, 1.18)
	College and above	19	202	0.30 (0.15, 0.60)	0.26(0.12,0.54)*
Child sex	Male	48	227	1	1
	Female	48	338	0.67 (0.43, 1.03)	0.63 (0.44, 1.14)
Household food security status	Food insecure	27	255	1	1
	Food secured	69	310	2.10 (1.30, 3.38)	1.96 (1.13, 3.39)*
Wealth index	Poorest	17	115	1	1
	Poor	10	122	0.55 (0.24, 1.26)	0.48 (0.32, 1.95)
	Middle	17	111	1.04 (0.50, 2.13)	1.03 (0.60, 3.12)
	Rich	22	146	1.02 (0.52, 2.01)	1.01 (0.65, 3.56)
	Richest	30	71	2.86 (1.47, 5.56)	2.72(2.01, 5.63)*

*Significantly associated factors at value of *p* ≤ 0.05, DDS, dietary diversity score; COR, Crude odds ratio; AOR, Adjusted odds ratio.

## Discussion

In this study, the overall prevalence of DBM among mother–child pairs in Bahir Dar City was 14.5% (95% CI (12.8 15.7%), which is close to the study conducted in Palestine (15.7%) (9). However, the results of this study were lower than studies conducted in Ethiopia (23%) (7), West Java, Indonesia (21.2–30.6%) (8) (55), Manipal Karnataka (23%) (56), South Karnataka, India (27.4%) (57), and rural district peninsular Malaysia (29.6%) (58). This variation could potentially be attributed to factors such as socioeconomic status, the geographical location of the study (urban or rural), and the utilization of different cutoff points and criteria for defining malnutrition. These factors may contribute to the observed discrepancies in the findings.

Conversely, the prevalence in the current study was higher than other studies study done in Addis Ababa and the rural district of Kersa (9%) (12), Tanzania(11·3%) (59), rural areas of Western Kenya (3%) (60), Bangladesh (5.5, 6.3%) (61, 62), Nepal (6.6%) (63), Brazil (2.6%) (44), India (7 to 12.3%) (64, 65), Peru (7%) (66), South and Southeast Asia (12%) (67). These differences could be attributed to the economic development of the population and the process of urbanization, which are associated with dietary changes and the prevalence of obesity. Additionally, it is worth noting that this study examines various forms of malnutrition, while the study in Kenya focuses specifically on the double burden of malnutrition in the context of stunted children with overweight mothers. This difference in study design and focus may contribute to variations in the results obtained.

The present study outlined that maternal educational status was negatively associated with the double burden of malnutrition. Mothers with higher educational status (college and above) were 74% less likely to be associated with DBM as compared to illiterate mothers (unable to read and write). This finding is in line with studies conducted in Ethiopia (7), South and Southeast Asia (67), Pakistan (43), and Peninsular Malaysia (68). One possible explanation for this discrepancy is that educated mothers have the ability to read, learn, and comprehend health and nutrition-related information, which can lead to positive changes in feeding behaviors (27). Educated mothers tend to possess greater knowledge and awareness about diets and lifestyle factors, enabling them to make more informed decisions regarding their own and their children’s nutrition.

The wealth index is also another factor associated with DBM. Participants who were in the richest wealth category had higher odds of DBM compared with the poorest categories. The finding was supported by studies conducted in Addis Ababa and the rural district of Kersa (12), Kenya (47), Indonesia (55), Bangladesh (62), Peninsular Malaysia (68), Tanzania (59, 69), South and Southeast Asian (67), Pakistan (43), and India (64). This could be attributed to the fact that households in the highest wealth index category may undergo a dietary transition, leading to increased consumption of energy-dense foods, a lack of physical exercise, and a higher intake of soft drinks and fatty foods, but poor in nutrients (64). These factors contribute to a higher risk of overweight and obesity among individuals in these households.

Children who had high DDS had lower odds of DBM. This finding was supported by studies done in Tanzania (69), Indonesia (55), and Malaysia (70). DDS refers to the number of food groups consumed in a given time, often in 24 h, and that emerged as a measure of the nutritional adequacy of a given individual (71). This could be because a lower dietary diversity score (DDS) relates to inadequacy of nutrient quality and quantity as well, which significantly exacerbates micronutrient deficiencies, which in turn contribute to chronic malnutrition and stunting. Insufficient consumption of essential nutrients hampers proper growth and development, resulting in long-term effects on the physical and cognitive well-being of individuals.

Notably, this study showed that household food security is often associated with increased odds of double burden of malnutrition (DBM), which is reported to be the opposite of most of the studies conducted worldwide (44, 55, 72). The consumption of high amounts of saturated fat, sugar, and refined foods, coupled with a limited intake of fiber-rich foods and a lack of physical activity, can increase the risk of overweight or obesity in mothers. Conversely, under nutrition in children can arise not only from inadequate food intake but also from poor hygiene and sanitation practices. These factors can make children more susceptible to recurrent infections, which can affect the absorption and utilization of nutrients in their bodies (73).

## Conclusion

In this study, the odds of experiencing the double burden of malnutrition among mother–child pairs were found to be lower compared to the findings reported in the EDHS 2016 (Ethiopian Demographic and Health Survey) report. In this study, educational status, wealth index, child dietary diversity score, and household food security status were identified as significant factors associated with the double burden of malnutrition. The study also highlights the importance of educating parents and caregivers about the benefits of food diversity for their children. In the future, researchers plan to conduct a prospective follow-up study on the double burden of malnutrition, considering additional variables such as micronutrient deficiencies and common non-communicable diseases. This will provide a more comprehensive understanding of the factors contributing to the double burden of malnutrition and its long-term implications.

## Limitations and strengths of the study

The study examined both dimensions of the double burden of malnutrition, which involve scenarios of undernourished mothers with over nourished children and over nourished mothers with undernourished children. However, there are certain limitations to consider. Firstly, the nutritional status of the mothers was assessed solely based on BMI (Body Mass Index), which may not provide a comprehensive representation of their overall nutritional status. Secondly, since the study was cross-sectional in nature, it did not establish a causal relationship between the variables examined. These limitations should be taken into account when interpreting the findings of the study.

## Data availability statement

The original contributions presented in the study are included in the article/supplementary material, further inquiries can be directed to the corresponding author.

## Ethics statement

The studies involving humans were approved by Bahir Dar University, College of Medicine and Health Science. The studies were conducted in accordance with the local legislation and institutional requirements. Oral informed consent was obtained from each rspondents/participating mother.

## Author contributions

SM: Writing – original draft, Writing – review & editing. DB: Writing – original draft, Writing – review & editing. YM: Writing – original draft, Writing – review & editing. ZG: Writing – original draft, Writing – review & editing. MB: Writing – original draft, Writing – review & editing.
